# Buckling and Post-Buckling Behavior of Uniform and Variable-Density Lattice Columns Fabricated Using Additive Manufacturing

**DOI:** 10.3390/ma12213539

**Published:** 2019-10-29

**Authors:** Aamer Nazir, Ahmad Bin Arshad, Jeng-Ywan Jeng

**Affiliations:** 1 High Speed 3D Printing Research Center, National Taiwan University of Science and Technology, No. 43, Section 4, Keelung Road, Taipei 106, Taiwan; M10703827@ntust.edu.tw; 2 Department of Mechanical Engineering, National Taiwan University of Science and Technology, No. 43, Section 4, Keelung Road, Taipei 106, Taiwan

**Keywords:** additive manufacturing, lattice structure, design and optimization, unit cell morphology, critical buckling load, variable density

## Abstract

Lattice structures are known for their high strength-to-weight ratio, multiple functionalities, lightweight, stiffness, and energy absorption capabilities and potential applications in aerospace, automobile, and biomedical industry. To reveal the buckling (global and local) and post-buckling behavior of different lattice morphologies, both experimental and simulation-based studies were carried out. Additionally, a variable-density lattice structure was designed and analyzed to achieve the optimal value of critical buckling load. Latticed columns were fabricated using polyamide 12 material on multi jet fusion 3D printer. The results exhibited that the buckling in lattice columns depends on the distribution of mass, second moment of inertia I, diameter and position of vertical beams, number of horizontal or inclined beams, and location and angle of the beams that support the vertical beams. The number of horizontal and inclined beams and their thickness has an inverse relation with buckling; however, this trend changes after approaching a critical point. It is revealed that vertical beams are more crucial for buckling case, when compared with horizontal or inclined beams; however, material distribution in inclined or horizontal orientation is also critical because they provide support to vertical beams to behave as a single body to bear the buckling load. The results also revealed that the critical buckling load could be increased by designing variable density cellular columns in which the beams at the outer edges of the column are thicker compared with inner beams. However, post-buckling behavior of variable density structures is brittle and local when compared with uniform density lattice structures.

## 1. Introduction

Cellular structures have become an essential application of additive manufacturing (AM) methodologies as AM methods provide complexity free fabrication of intricate geometries at micro, meso, and macroscale. Additive manufacturing is defined as a “process of joining materials to make objects from 3D model data, usually layer upon layer, as opposed to subtractive manufacturing methodologies, such as traditional machining” by the Additive Manufacturing (AM) Technology Standard F2792-10 [1]. Additive manufacturing allows multiple benefits over traditional manufacturing such as fabrication of complex geometries without the associated increase in cost, low material wastage, no tooling requirements, personalization and customization, and reduction in cost and time of manufacturing [2,3]. Although the aforementioned characteristics of AM technologies make it preferable over traditional manufacturing in some use cases, there are also several challenges in AM that restrict its extensive use in the manufacturing industry [3].

Cellular solids are constructed by an interlinked network of structural components such as beams, plates, trusses, and unit cells. They are found quite frequently in nature [4]. Cellular structures can also be defined as mesostructures that have a characteristic length from 0.1 mm to 10 mm. Some mesostructures are manufactured by stochastic method, for e.g., foaming. The other type of mesostructure is the designed cellular structure [5]. Due to the multidimensional characteristics of these cellular structures, they have generated a great interest in the industry because of their potential applications. The primary advantage of cellular structures is that the material is added only where it is required. This is leading to innovation in the design and manufacturing methods of the cellular structures because the use of these structures allows saving expensive functional materials, reduction in build time, and energy consumption. At the same time these structures offer high performance; high stiffness to weight ratio [6,7,8,9,10]; excellent energy absorption features [11,12,13]; low heat conductivity [14]; and thermal and acoustic insulation for aerospace structures [15], automotive parts, engineering, and medical products [7,16].

Cellular structures have been used extensively in the aerospace industry due to their high strength-to-weight ratio. This leads to an increase in the efficiency of the aircraft because of an increase in the performance-to-weight ratio [17]. The maximization of surface area and high strength-to-weight ratio is of particular use to the biomedical industry because the use of cellular structures with aforementioned properties allows a better fixation of biomedical implants with the body by allowing for improved ingrowth of human tissue [18]. Cellular structures are also used to manufacture metallic parts. In the automotive industry, cellular structures are used to make heat exchangers, sound absorbers, filters, acoustic dampers, lightweight structures, and catalyst support, thus improving the comfort, safety, and efficiency of the vehicle [19]. In some cases, lattice structures have been exploited by aerospace engineers to construct stiff, robust, tall but ultralight columns, employed in aerospace crane arms [20], aerospace masts [21], deployable columns [22], and solar sails. However, there are many challenges still that need to be addressed in cellular structures.

Buckling instability is a serious problem that leads to the premature failure in lattice structures that are under the action of compressive load. The induced compressive stresses at the point of buckling failure are less than the ultimate compressive stress of the material that is used to manufacture that structure [23]. The Buckling of shells [24], planes, and beams [25,26] has been extensively investigated in recent years. Lattice structures can be either beneficial when changing the lattice parameters to improve the static and dynamic properties of the structure, or they may be harmful if lattice design is inappropriate, and parameters are used which may cause premature failure due to enhanced crack propagation. [27]. Consequently, it is necessary to determine the optimal parameters of lattice structure for a specific application [28,29]. Qianqian et al. [30] developed theoretical and finite element methods for analyzing one-dimensional lattice truss composite structures under uniaxial compression and found that the buckling modes significantly depend upon the length of the column. Magnucki et al. [31] derived an analytical solution for investigating critical buckling load of a unidirectional isotropic beam made of porous material. Magnucka-Blandzi [32] performed the buckling and bending analysis on a porous plate under the action of buckling force and uniformly distributed load. Overvelde et al. [33] investigated the effect of structural shape on the buckling behavior of a two-dimensional lattice structure and revealed that the morphology of structure affects the buckling behavior of the soft porous system. In another study [34], authors studied the effect of lattice structure shape, plate thickness, and different boundary conditions on the buckling behavior of functionally graded porous plate. In a more recent study, Tang. et al. [35] evaluated the effect of different porosity distribution patterns on buckling behavior of two-directionally porous beams and concluded that the porosity distribution patterns significantly affect the critical buckling load. Another study [36] revealed that the lattice pore size also affects the buckling behavior of the lattice structure significantly.

Most of the prior work done on lattice structures has been focused on investigating the compressive properties [37,38,39] of various lattice shapes; however, a limited number of studies have been investigated the buckling behavior of a very few lattice morphologies. Moreover, buckling analyses of the samples having circular and elliptical holes/pores have been carried out [35,36,40,41,42,43,44]. However, the effect of unit cell morphology and size on the critical buckling load and post-buckling behavior has not been explored yet. Therefore, it is necessary to conduct an experimental and analytical study to analyze the buckling behavior of cellular columns designed using different lattice morphologies.

This study aims to investigate the effect of unit cell morphology on buckling and post-buckling behavior of the column constructed with various lattice morphologies, as shown in Table 1. Samples were additively manufactured, and uniaxial compression tests were performed on samples having different lattice morphologies but where the mass, volume, and dimensions of all samples remained constant. Furthermore, the best performing lattice morphologies were further analyzed using finite element analysis (FEA). Finally, the effect of variable density lattice morphologies on buckling behavior was investigated, and an optimal point was determined in order to achieve the highest value of critical buckling load. This paper is organized into four sections. A brief introduction of additive manufacturing, cellular structures, and buckling behavior of structures is given in Section 1. Section 2 explains the experimental method which includes design of lattice morphologies and samples, AM of samples, and the procedure of mechanical testing. Experimental results are discussed in Section 3. In addition, the best performing structures are further optimized and discussed in this section. Finally, the conclusion of this study is summarized in Section 4.

## 2. Materials and Methods

### 2.1. Design of Unit Cell and Samples

PTC Creo software was used to design different lattice morphologies and then the entire sample. Cubic structure is the simplest one in which the beams are oriented only in horizontal and vertical directions, and the unit cell dimensions are the same in all three axes. Based on the importance of lattice beams at different orientations [45], the remaining unit cells were designed to investigate the buckling behavior by placing the materials at different orientations. For the inner inclined cubic structure, the beams are oriented in horizontal, vertical, as well as the inclined (45°) plane inside the unit cell. The inclined cubic structure has a similar orientation of inner inclined cubic plus the inclined beams (45°) which are oriented on all faces of the unit cell. Face inclined beams have all features of simple cubic plus the inclined beams on all six faces of the unit cell. Octet Truss is already well defined in many research studies [4]. Vertical inclined has vertically oriented beams on four edges and center of the unit cell plus the inclined beams intersecting the center vertical beam at the center point. Vertical inclined (m1) is the modified form of vertical inclined structure. In this structure, all vertical beams have radius of 2 mm whereas the inclined beams have 1.22 mm radius. The unit cell sizes and beam thickness were adjusted to keep all samples uniform in weight, volume, and external dimensions.

### 2.2. Additive Manufacturing of Samples

Polyamide (PA12) powder was used for additive manufacturing of lattice structured column samples. Polyamide 12 (PA12) is a well-known plastic normally used for the injection molding of parts intended for engineering applications. Due to its excellent properties, this material has already been used in wide range of industries including aerospace, automotive, medical, defense, and environmental for prototyping as well as completely functional parts. The printed samples of various morphologies were tested in uniaxial compression to investigate the buckling strength, particularly critical buckling load. Some of the 3D printed samples are shown in the last row of Table 1.

All samples were 3D printed at vertical orientation on a high-speed multi jet fusion (MJF) 4200 series 3D printer [46]. This printer used three dual-agent printheads that have 31,680 thermo-bubble nozzles each to deposit 10 µm drops of agents on the build platform where the layer of PA12 powder is already placed. The MJF process can achieve a printing speed of 4115 cm^3^/h. An IR light is used to apply the energy to fuse the material to make the object. Using MJF process, a minimum feature size of 1 mm and a minimum gap of 1 mm can be printed. For each lattice morphology, three samples were fabricated and experiment was performed; the complete data of samples height (h), unit cell size (u), beam radius (r), volume (V), and mass (M) are shown in Table 1, while the same width (w = 21.2 ± 0.1 mm) was used for all samples. All samples have same external dimensions, mass, and volume.

### 2.3. Mechanical Tests

**Figure 1:**
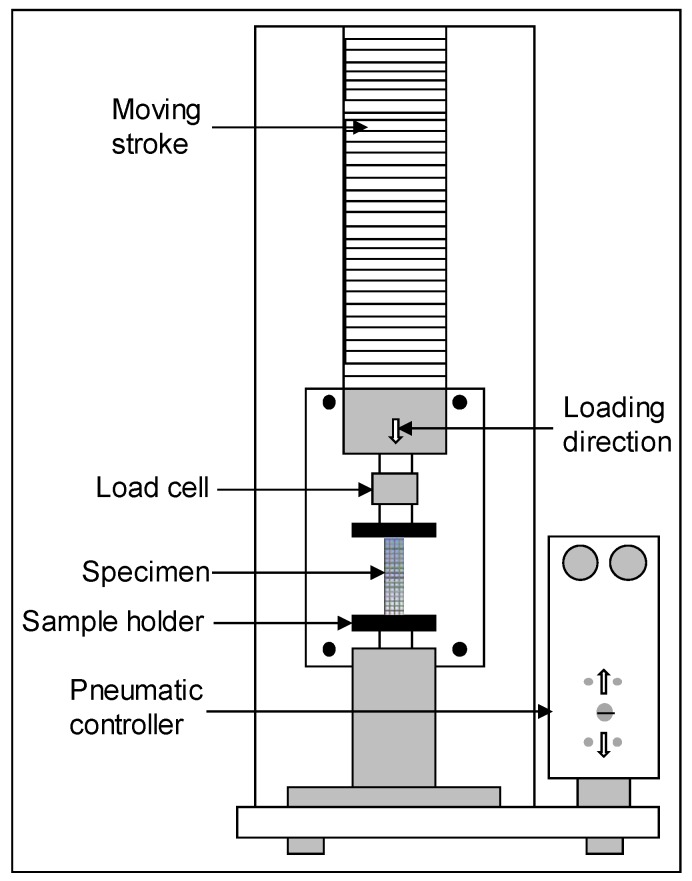
The Schematic diagram of the compression testing machine. The lattice structured compressive specimens were fixed in the sample holder and subjected to a displacement-controlled compressive load.

In order to determine the mechanical properties of PA12 material, the dog bone samples (ASTM D638 type 4) were printed and tested on MTS universal testing machine. The material properties are listed in Table 2. The Young’s modulus was calculated by testing the tensile samples while the density and Poisson’s ratio were obtained from vendor’s data [46]. The uniaxial compression tests were performed on the MTS universal testing machine (MTS Systems Corporation, Eden Prairie, MN, USA) with a 10 KN load cell, at a test speed of 2 mm/min. An established and widely accepted procedure [36,42,43,47,48] of axial compression was followed to test the compression samples. Once the buckling happened in the sample, the slope of the load-displacement curve started decreasing; and the test was stopped. Force and displacement data were continuously recorded using the software Testworks 4.0. A schematic diagram of compression testing setup is shown in Figure 1.

## 3. Results and Discussion

**Figure 2:**
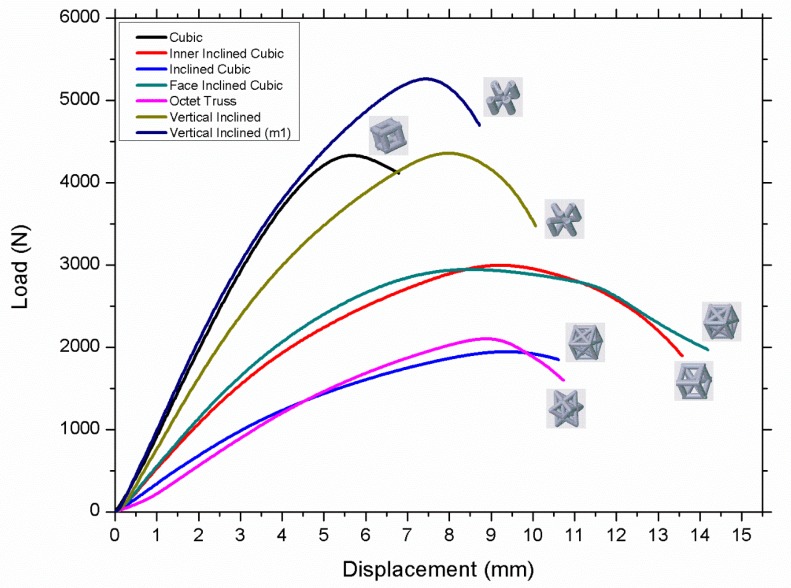
Experimental load-displacement curves showing the effect of the lattice morphology on the critical buckling load.

Figure 2 shows the critical buckling load (Pcr) versus different lattice morphologies, where the sample height, volume, and mass remains constant. It can be seen that the vertical inclined (m1) lattice topology has the maximum critical buckling load, followed by vertical inclined, cubic, inner inclined cubic, face inclined cubic, octet truss, and inclined cubic respectively. Table 3 shows that the volume and mass of all samples remain same, i.e., the change in critical buckling load is significantly influenced by cellular morphology of the lattice column which is different for each sample. 

**Figure 3:**
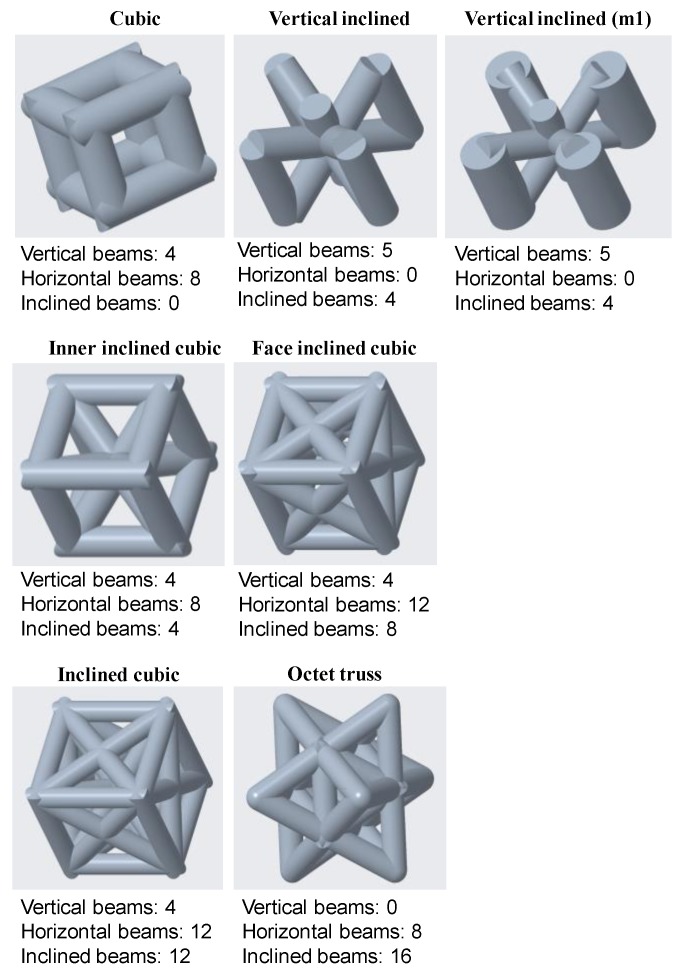
The number of beams oriented in vertical, horizontal, and inclined directions of different lattice morphologies.

The results shown in Figure 2 can be categorized based on the number of vertical, horizontal, and inclined beams used in the unit cell design of each morphology as shown in Figure 3. It was revealed that the orientation of beams also affects the buckling resistance of the structure. The vertical inclined, vertical inclined (m1) and cubic structure had the lowest number of beams oriented horizontally or in inclined direction, so they have a high Pcr value compared to their counterparts. Conversely, the octet truss and inclined cubic structure have the lowest number of vertical and highest number of horizontal and inclined beams and have the least resistance to buckling as shown in Figure 6.

**Figure 4:**
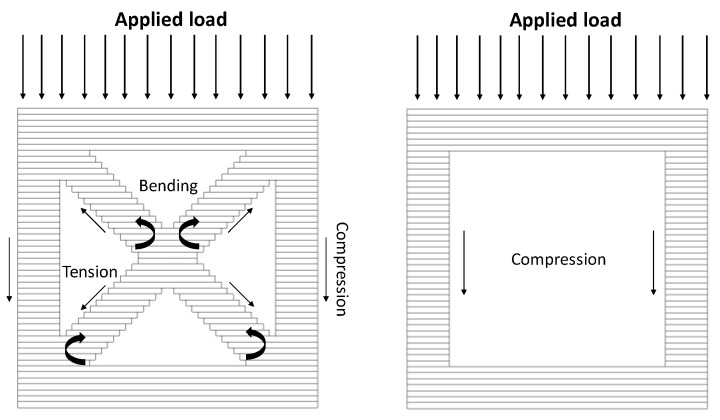
The figure shows the deformation behavior of additively manufactured structure with and without inclined beams.

In the cross-section with no inclined beams, the force is transmitted vertically downwards; i.e., the vertical members are in compression (see Figure 4 right). The horizontal members stop the structure from buckling earlier, so they are both in compression and tension depending on their location in the structure. In the middle of the structure, the horizontal beams would be in tension as they would prevent the structure from being pushed outwards under the effect of the compressive force from the top. For the structures which have inclined beams, it becomes difficult to segregate all the stresses since they undergo multiple types of stresses at the same time. The vertical beams are in compression, which causes compressive stress that would induce a moment in the inclined beams. Consider two inclined beams in the middle of two vertical beams (see Figure 4 left). The horizontal beams can be neglected in this case by replacing them with the boundary conditions. When the right beam is in compression, it bends the inclined beam, which joins it at the top, downwards. The vertical beam on the left also bends the inclined beam that is joined to it at the bottom, downwards. This causes two opposite bending moments in the same beam. However, this beam is supported in the middle by the other inclined beam which is also undergoing a similar stress condition. Due to this, the bending modes of both inclined beams have an intersection point at the location where both beams intersect. This intersection point is the concentration of the highest stress in the unit cell. Apart from bending stresses, these beams are also in tension and compression due to the structure stretching outwards when it is compressed from top. It also adds to the amount of stress at the intersection point.

**Figure 5:**
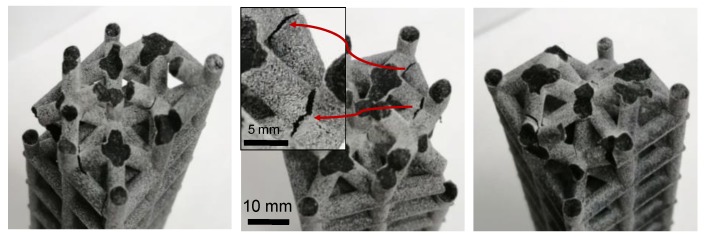
The figure shows an inner inclined structure in which the outer beams failed from the middle point whereas the inner inclined beams failed at the point of intersection.

The outer beams in Figure 5 have failed from the middle, indicating failure due to local buckling of the column. It is apparent from the half-length stubs in the outer columns. The inclined beams in the inner direction have failed from the intersection points. The cracks can also be seen as the beams began to shear away from the intersection point.

**Figure 6:**
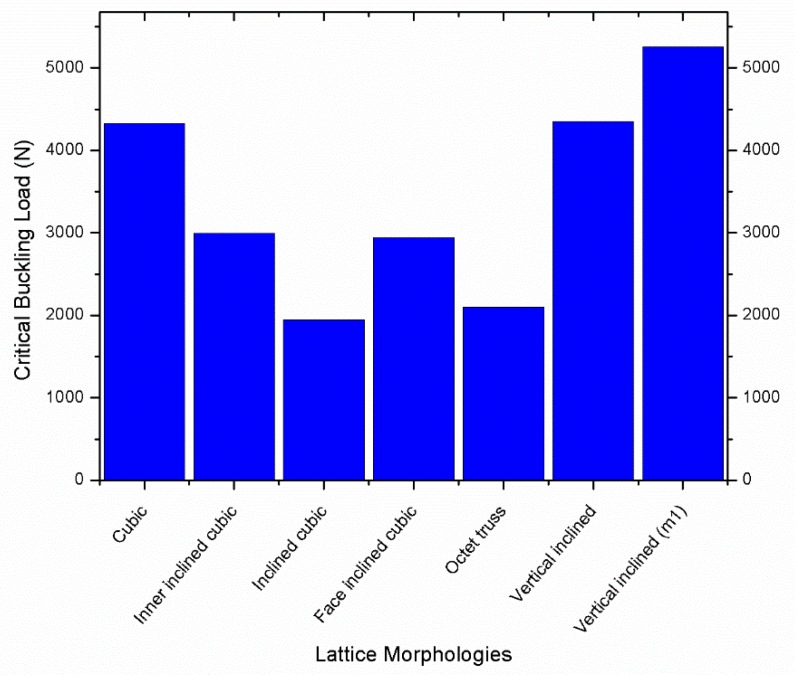
Critical buckling load of different lattice morphologies.

From the above discussion, it can be concluded that the structures which have higher number of inclined beams are least resistant to buckling loads because inclined beams may fail due to tension, bending, and shear; in particular, for additively layer-by-layer manufactured samples, even the shear stresses at layer interfaces may also affect the buckling behavior of samples. Figure 6 shows the critical buckling load for each lattice morphology.

**Figure 7:**
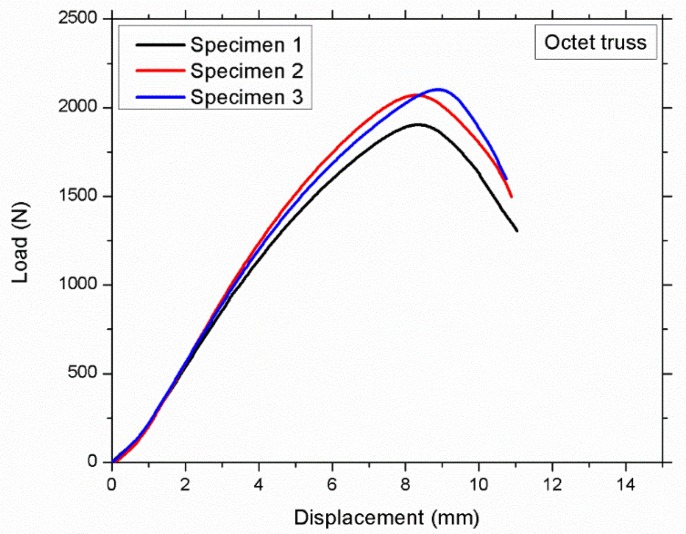
Octet truss structured specimens having mass of 30.92, 31.58, and 30.83 g, respectively, showing minor variations in critical buckling load.

There are small variations in the mass of different lattice structures, as shown in Table 3; however, these minor changes are not the main reason for significant change of buckling load of different samples. Figure 7 shows Pcr versus displacement of octet structured specimens that have minor variations in mass which causes minor variations (1903 N, 2070 N, and 2100 N respectively) in Pcr value but these variations in mass cannot effect Pcr value significantly.

Euler’s formula is used for solid beams

(1) $$P_{cr}=\frac{\pi^{2}EI}{{\left(kL\right)}^{2}}$$

where E is the modulus of elasticity, I is the second moment of inertia, column length L, and k is length factor. In case of solid column, the mass distribution is constant, so the value of I is also assumed as constant.

For this study, E, k, and L remain constant, and only I changes with the change in lattice morphology of structure, i.e., it is possible to change I by shaping the cross-section of the object.

(2) $$P_{cr}\propto I$$

**Figure 8:**
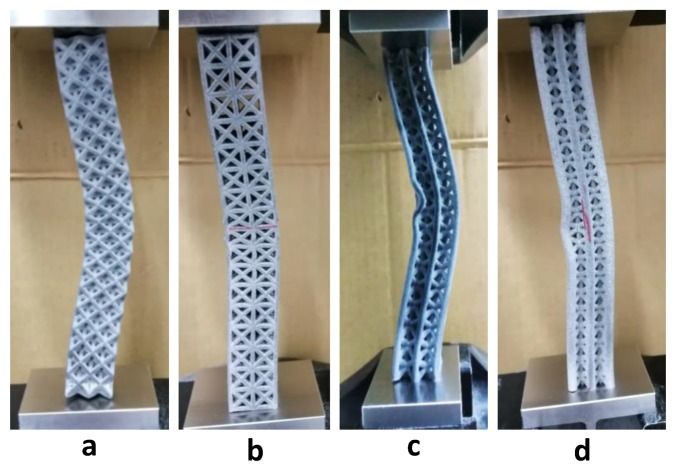
Buckling behavior of (**a**) octet truss and (**b**) inclined cubic structure (**c**) vertical inclined structure (**d**) vertical inclined (m1) structure.

From the above Euler’s formula, Pcr is directly proportional to I, i.e., the value of Pcr can be increased by varying the value of I. This can be achieved by placing more material as far from the neutral axis as possible [49]. For this study, different lattice structures have different mass distributions; therefore, have different values of I for all structures. The vertical inclined (m1) structure has the highest value of Pcr because it has thicker outer beams, i.e., more mass is distributed far from the neutral axis. The vertical beams are supported by angular beams that may allow better transmission of forces in vertical inclined (m1) and vertical inclined structure. However, these angular beams may not increase the Pcr significantly because both vertical inclined and cubic structure have similar value of Pcr. The performance of inner inclined cubic and face inclined cubic is lower than cubic and vertical inclined structure because there is more mass distributed near the central axis and also mass is distributed at angular orientation in face inclined cubic structure. Due to such distribution, the diameter of vertical beams has reduced, therefore reducing the value of Pcr. Octet truss and inclined cubic structures performed worst because of either lowest diameter of outer vertical beams or absence of outer vertical beams. However, the post-buckling behavior of these structures is more ductile when compared with high performing structures (such as vertical inclined and vertical inclined (m1) as shown in Figure 2). The absence of vertical beams allows the structure to buckle without breaking the material as shown in Figure 8a,b. Conversely, the structures with more material distributed in vertical orientation (see Figure 8c,d) have local buckling behavior dominated over global buckling. In addition, the structures that have more mass distributed in vertical orientation buckled in a brittle manner when compared with their counterparts, which have uniform material distribution, e.g., octet and inclined cubic structures. The post-buckling behavior of each lattice morphology can be understood by observing the post-buckling curves shown in Figure 2.

**Figure 9:**
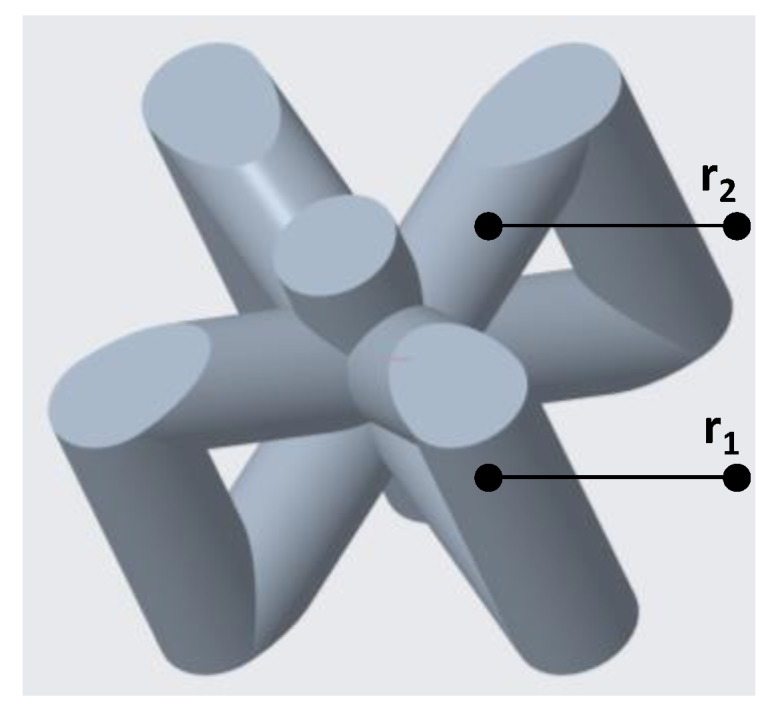
A vertical inclined unit cell is shown in which r_1_ and r_2_ are the radii of vertical and inclined beams respectively.

Based on the results mentioned in Figure 2, the best performing vertical inclined structure was chosen for further analysis and optimization. A vertical inclined unit cell is shown in Figure 9 in which r_1_ denotes the radius of vertical beams while r_2_ is the radius of supporting inclined beams.

**Figure 10:**
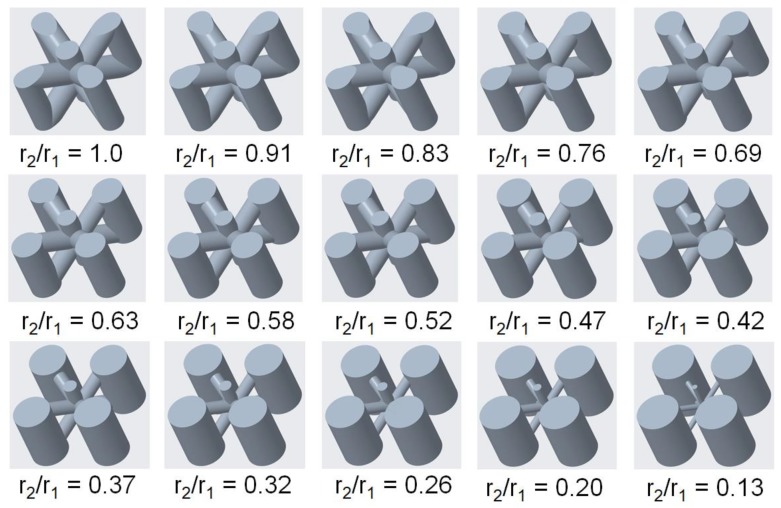
Vertical inclined cellular unit cell with different ratios of r_1_ and r_2_.

For this study, the results revealed that the vertical inclined (m1) structure performed best in terms of buckling load, i.e., the Pcr can be improved by varying the values of r_1_ and r_2_ while the total mass and volume of the sample remain same. The results also exhibited that Pcr increases by increasing the radius of vertical beams while reducing r_2_. Therefore, an optimal point needs to be searched at which the Pcr has a maximum value. Finite Element Analysis (FEA) using ANSYS eigenvalue buckling solver was performed on variable-density cellular columns having different ratios of r_2_ and r_1_ as shown in Figure 10. The values of r_1_ and r_2_ for different values of r_2_/r_1_ ratios are mentioned in Table 4. The density (1.01 g/cm^3^), Young’s modulus (1250 MPa), and Poisson’s ratio (0.33) were used to define the material for FE analysis. The cellular column is fixed in translation and rotation except the longitudinal displacement (y ≠ 0) which is free. The bottom end of the specimen was restrained in both longitudinal and lateral displacement as well as rotation. A uniaxial load was applied at the top end to obtain eigenvalues from which critical buckling load can be calculated by multiplying eigenvalue with the applied load.

**Figure 11:**
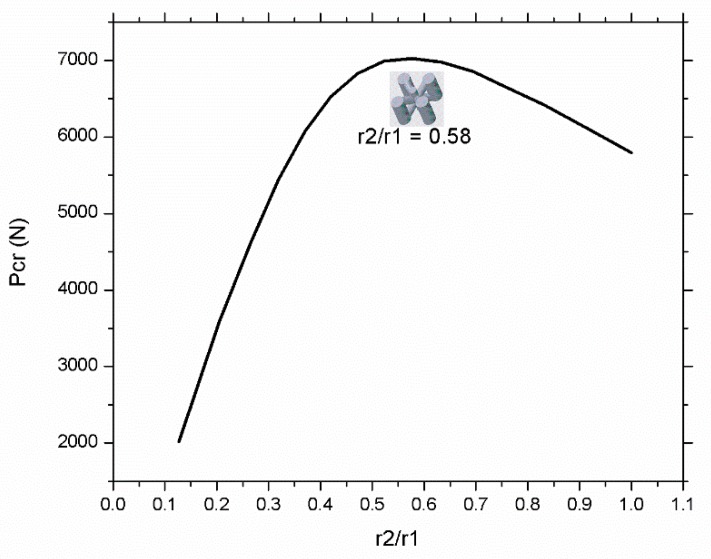
Showing the effect of r_2_/r_1_ ratio on critical buckling load and the optimal value of Pcr.

Figure 11 illustrates the relationship between r_2_/r_1_ ratio and the critical buckling load. The r_2_/r_1_ ratio decreases with the decrease of inclined beams rigidity by reducing the radius of these diagonal beams. The Pcr increases with the increase of r_2_/r_1_ ratio until a critical point when r_2_/r_1_ becomes equivalent to 0.58 at which the Pcr has a maximum value. It is found that vertical beams are more important when compared with horizontal or inclined beams; however, material distribution in inclined or horizontal orientation is also crucial because it provides support to vertical beams to behave as a single body to bear buckling load. Additionally, it is found that the buckling behavior of variable density structure is better than uniform density structure while using same mass and volume. This result can be validated using experimental results (see the value of Pcr of experiments 6 and 7 in Figure 2 and Table 3) in which the variable-density vertical inclined (m1) structure has significantly higher value of Pcr compared to its uniform density counterpart.

## 4. Conclusions

In this study, the effect of the unit cell morphology on the critical buckling load and post-buckling behavior of uniaxial compressive columns is investigated. For this purpose, uniaxial compressive tests were performed on additively manufactured samples constructed using different lattice morphologies. The present study has highlighted the importance of selecting an optimal lattice morphology and variable density lattice structures for a particular object while considering the buckling failure of the object.

It can be concluded that the buckling in cellular structures depends on the distribution of mass, second moment of inertia I, diameter and position of vertical beams, number of horizontal or inclined beams, and location and angle of the beams that support the vertical beams. It was found that the vertical inclined (m1) and vertical inclined structures have high resistance to buckling, whereas, inclined cubic and octet truss structures have least resistance to buckling. The number of horizontal and inclined beams and their thickness has an inverse relation with buckling; however, this trend changes after approaching a critical point. The vertical inclined, vertical inclined (m1) and cubic structure have the lowest number of beams oriented horizontally or in inclined direction, so they have a high Pcr value compared to their counterparts. Conversely, the octet truss and inclined cubic structure have the lowest number of vertical and highest number of horizontal and inclined beams and have least resistance to buckling.

It is revealed that vertical beams are more crucial for buckling case, when compared with horizontal or inclined beams; however, material distribution in inclined or horizontal orientation is also critical because it provides support to vertical beams to behave as a single body to bear the buckling load. It is found that the buckling resistance can be increased by placing more material as far from the neutral axis as possible. It can be achieved by designing variable density structures which were also investigated in this study. For the same total mass, volume fraction, and dimensions of the cellular column, the Pcr value can be increased by designing variable density cellular columns in which the beams at the outer edges of the column are thicker compared with inner beams. However post-buckling behavior of variable density structures is brittle and local when compared with uniform density lattice structures.

This study also revealed that a lightweight cellular column could be designed by optimally distributing the mass within design domain that can bear the same amount of load (same Pcr value) compared with other heavier count-parts. This study is limited to only uniaxial compression latticed columns. A further parametric simulation study on the effect of the unit cell shape on the critical buckling load is suggested for future work.

## Figures and Tables

**Table 1:** Different types of cellular morphologies investigated in this study.

	Cubic	Inner Inclined Cubic	Inclined Cubic	Face Inclined Cubic	Octet Truss	Vertical Inclined	Vertical Inclined (m1)
Unit Cell	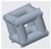	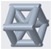	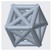	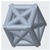	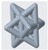	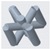	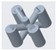
Top View of Sample	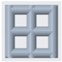	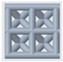	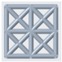	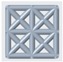	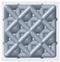	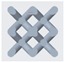	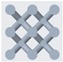
Designed Samples	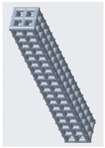	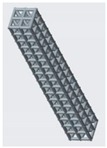	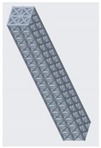	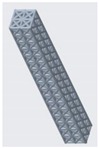	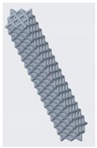	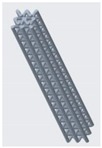	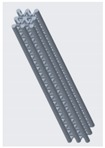
3D Printed Samples	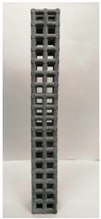	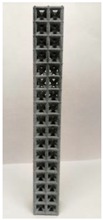	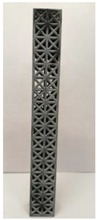	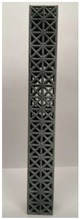	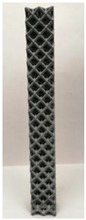	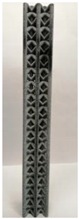	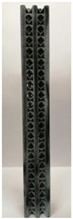

**Table 2:** Material properties of PA12 polymer.

Density (g/cm^3^)	Young’s Modulus (MPa)	Poisson’s Ratio
1.01	1250	0.33

**Table 3:** Design of experiment, parameters of samples, and results.

Experiment No.	Lattice Morphology	Sample Height: h (mm)	Unit Cell Size: u (mm)			Beam Radius: r (mm)	Volume: Calculated V (mm3)	Mass: M Measured (grams)	Critical Buckling Load (Pcr)(N)
**1**	Cubic	161.2 ± 1	8.72	8.72	8.72	1.962	32,055	29.2	4330
2	Inner Inclined Cubic	161.2 ± 1	9.24	9.24	9.24	1.35	32,008	29.4	2994
3	Inclined Cubic	161.2 ± 1	9.65	9.65	9.37	0.94	31,593	28.9	1943
4	Face Inclined Cubic	161.2 ± 1	9.45	9.45	9.35	1.145	31,971	29.4	2944
5	Octet Truss	161.2 ± 1	9.55	9.55	9.55	1.05	32,018	30.8	2101
6	Vertical Inclined	161.2 ± 1	9.1	9.1	9.48	1.5	32,290	29.5	4356
7	Vertical Inclined (m1)	161.2 ± 1	8.6	8.6	8.1	2, 1.22	32,014	29.2	5260

**Table 4:** The buckling load for different r_2_/r_1_ ratio while the volume and mass remains the same for all samples as indicated in first and second column from the left.

Volume (mm^3^)	Mass (g)	r_1_ (mm)	r_2_ (mm)	r_2_/r_1_	Pcr (N)
41,545	41.96	1.44	1.44	1.000	5796
41,549	41.964	1.55	1.412	0.911	6129
41,533	41.948	1.66	1.38	0.831	6421
41,430	41.844	1.77	1.342	0.758	6654
41,541	41.956	1.88	1.305	0.694	6857
41,556	41.971	1.99	1.261	0.634	6982
41,543	41.958	2.1	1.211	0.577	7028
41,567	41.983	2.21	1.156	0.523	6990
41,558	41.973	2.32	1.093	0.471	6832
41,532	41.948	2.43	1.021	0.420	6529
41,550	41.966	2.54	0.94	0.370	6076
41,554	41.97	2.65	0.845	0.319	5438
41,536	41.951	2.76	0.73	0.264	4602
41,543	41.959	2.87	0.586	0.204	3574
41,542	41.957	2.98	0.378	0.127	2015
